# EFFECTS OF A CANCER DIAGNOSIS ON ECONOMIC PROSPERITY IN THE LONG RUN: EVIDENCE FROM LINKED SURVEY AND CREDIT DATA

**DOI:** 10.1093/geroni/igae098.0092

**Published:** 2024-12-31

**Authors:** John Warren, Amanda Thomas, Eric Grodsky, Chandra Muller

**Affiliations:** University of Minnesota, Minneapolis, Minnesota, United States; University of Minnesota, Minneapolis, Minnesota, United States; University of Wisconsin Madison, Madison, Wisconsin, United States; The University of Texas Austin, Austin, Texas, United States

## Abstract

Although 40% of Americans will experience cancer in their lifetime, little is known about the long-term impacts of cancer diagnosis on debt, wealth, and financial precarity later in life. Using data from High School and Beyond (HSB) —a large (n~25,500) nationally representative study of high school students followed from 1980 through 2022—linked to consumer credit records (2004-2023), we estimate changes in debt, credit, and other economic measures resulting from after a cancer diagnosis. We also consider how these processes vary across demographic, educational, and family background groups. Approximately 13,900 individuals from the original cohort responded to the 2021/2022 survey, with more than 1,000 indicating a lifetime diagnosis other than skin cancer; another 490 cohort members died from various forms of cancer by 2021/2022. The most common cancers reported were breast (n=350) and prostate/vaginal (n=150 each) with more than one-third diagnosed in early adulthood (≤ 45 years old). Preliminary results using cross-sectional measures of financial well-being indicate reduced annual income earnings and heightened financial precarity as compared to otherwise similar people without a cancer diagnosis. With linked consumer credit data, we are well-equipped to expand knowledge about the effects of a cancer diagnosis on financial health by analyzing fluctuations in indicators such as credit score, medical and other forms of debt, bankruptcy, and estimated wealth in the months and years after a cancer diagnosis. Our analyses will model these outcomes, inclusive of key confounders (age, education, type of cancer, etc.) both for the full sample of demographic and socioeconomic subgroups.

